# Ablation Surgery for Atrial Fibrillation: *"Freeze it or Buzz it; Just do it and Cure it"*

**Published:** 2005-10-01

**Authors:** Patwardhan A M

**Affiliations:** Dept. of Cardio-Vascular and Thoracic Surgery, Seth G.S. Medical College and K.E.M. Hospital, Mumbai, India

**Keywords:** Atrial fibrillation, surgical ablation

## Abstract

Patients in normal sinus rhythm have lesser stroke rate, better functional class and quality of life than those in atrial fibrillation. Adding a surgical procedure to cure atrial fibrillation in patients needing correction of structural heart disease has been shown to be a safe option, which benefits the majority in restoration of sinus rhythm. Age is no bar to implement this option. The same does not hold true for lone atrial fibrillation. The affirm trial has shown that there is need for improved treatment strategies for patients in atrial fibrillation, although young patients were not represented in sizable proportion. There is need to develop curative treatment for patients with lone atrial fibrillation. And there are technological advances in the form of  ablative energy sources and hardware for applying these with minimal invasion. “Between tomorrow’s dream and yesterday’s regret is today’s opportunity”. Let’s make the best of it!

Why should one consider the surgical option to treat atrial fibrillation (AF)? The answer lies in the influence of this arrhythmia on the stroke rate, NYHA class and quality of life. In the United States, 2.2million people have AF and 75000 strokes occur in these patients each year [1]. The stroke rate varies between 2.5% to 5% per year under influence of associated risk factors, such as hypertension, diabetes mellitus, congestive heart failure and ischemic heart disease. James Cox [2] showed beyond any doubt that restoration of normal sinus rhythm (NSR) and obliteration of left atrial appendage, remarkably reduced the stroke rate. Vaturi et al [3] compared patients who had their mitral valve replaced, for their functional status with respect to rhythm. The patients were matched for age sex and time from valve replacement. The NYHA class for AF patients was 2.8 +/- 0.8 while that for NSR was 1.1+/- 0.7. The trans-mitral gradients were greater and the size of the atria was greater in patients in AF. Lonnerholm S. et al [4] showed that following the maze procedure the quality of life indices significantly improved as compared to pre-operative indices. All this data should make the physicians look for treatment options suitable for their patients in restoring sinus rhythm. And there can be no better opportunity for a surgeon for treating AF when the patient needs surgical correction of structural heart defect.

In this issue, Geidel S. et al [5] have shown that an intraoperative  radiofrequency approach is successful in 73% of patients with permanent AF who were above the age of 70 years. This was achieved with a low mortality and morbidity. The procedure time for performing the ablations is less than 15 minutes. This is enough evidence for the surgeons to treat permanent AF even in the elderly needing cardiac surgery.

The data from western countries shows that the incidence of atrial fibrillation rises with age and approximately 10 %  of patients above the age of 80 have AF. The Framingham study shows that AF is associated with a 1.5 fold greater risk of death for men and a 1.9 fold greater risk of death for women shortening men’s life by 18 years and women’s by 21 years [6]. The risk of stroke in patients over 70 years is increased 3 to 5 fold by  AF. The AFFIRM trial [7] has shown that there is no diference in survival or stroke rate in the rhythm control or rate control arms. The clinical outcomes in this trial have demonstrated the need for improved treatment strategies.  It follows that  truly curative therapies for treating atrial fibrillation need to be explored. As of now catheter based ablation therapy or surgical therapy holds promise in the patients who do not have structural heart disease but have permanent AF. Catheter ablation for paroxysmal AF has shown promise [8]. But Cox’s maze III  procedure which has been highly successful one has a reported 2% mortality [9]. Scahff et al [10] have reported an early mortality of 1.4% for the same procedure. The morbidity of any surgical procedure with the use of cardiopulmonary bypass is well known. It makes no sense to use a procedure even with any mortality to ablate lone AF. Therefore there was the need to develop a minimally invasive surgical procedure which would have no mortality and minimal morbidity and a high degree of success.   Thoracoscopic pulmonary vein isolation using microwave catheter has been done successfully [11]. The LA appendage can be stapled off with an endo GI stapler. Cryo catheters have been developed to ablate atrial myocardium in the cath lab and these can also be used  for minimally invasive surgical approaches. Thus the future holds great promise for cure for permanent AF without structural heart disease with minimal invasion. It is only through efforts of the medical community and with the support of the innovative industry that the promise can translate into reality.
